# Infant motor development in rural Vietnam and intrauterine exposures to anaemia, iron deficiency and common mental disorders: a prospective community-based study

**DOI:** 10.1186/1471-2393-14-8

**Published:** 2014-01-08

**Authors:** Thach D Tran, Tuan Tran, Julie A Simpson, Ha T Tran, Trang T Nguyen, Sarah Hanieh, Terence Dwyer, Beverley-Ann Biggs, Jane Fisher

**Affiliations:** 1 Research and Training Centre for Community Development, 39/255 Vong Street, Hai Ba Trung District Hanoi, Vietnam; 2 Centre for Women’s Health Gender and Society, Melbourne School of Population and Global Health, The University of Melbourne, Grattan Street, Parkville, VIC 3010, Australia; 3 Jean Hailes Research Unit, School of Public Health and Preventive Medicine, Monash University, Monash, VIC 3168, Australia; 4 Centre for Molecular, Environmental, Genetic & Analytic Epidemiology, Melbourne School of Population and Global Health, The University of Melbourne, Grattan Street, Parkville, VIC 3010, Australia; 5 Department of Medicine (RMH/WH), The University of Melbourne, The Royal Melbourne Hospital, 300 Grattan Street, Parkville, VIC 3050, Australia; 6 Murdoch Children’s Research Institute, Royal Children’s Hospital, 50 Flemington Road, Parkville, VIC 3052, Australia

**Keywords:** Infant development, Pregnancy, Common mental disorders, Micronutrient deficiencies, Vietnam

## Abstract

**Background:**

Antenatal anaemia, iron deficiency and common mental disorders (CMD) are prevalent in low- and middle-income countries. The aim of this study was to examine the direct and indirect effects of antenatal exposures to these risks and infant motor development.

**Methods:**

A cohort of women who were pregnant with a single foetus and between 12 and 20 weeks pregnant in 50 randomly-selected rural communes in Ha Nam province was recruited. Participants provided data twice during pregnancy (early and late gestation) and twice after giving birth (8 weeks and 6 months postpartum). The Edinburgh Postnatal Depression Scale was used at all four data collection waves to detect CMD (score ≥ 4). Maternal anaemia (Hb < 11 g/dL) and iron deficiency (ferritin < 15 ng/mL) were evaluated at early and late gestation. Infants’ motor development was assessed by the Bayley of Infant and Toddler Development Motor Scales (BSID-M) at the age of six months. Direct and indirect effects of the exposures on the outcome were examined with Path analysis.

**Results:**

In total, 497 of 523 (97%) eligible pregnant women were recruited and 418 mother-infant pairs provided complete data and were included in the analyses. The prevalence of anaemia was 21.5% in early pregnancy and 24.4% in late pregnancy. There was 4.1% iron deficiency at early pregnancy and 48.2% at late pregnancy. Clinically significant symptoms of CMD were apparent among 40% women in early pregnancy and 28% in late pregnancy. There were direct adverse effects on infant BSID-M scores at 6 months of age due to antenatal anaemia in late pregnancy (an estimated mean reduction of 2.61 points, 95% Confidence Interval, CI, 0.57 to 4.65) and CMD in early pregnancy (7.13 points, 95% CI 3.13 to 11.13). Iron deficiency and anaemia in early pregnancy were indirectly related to the outcome via anaemia during late pregnancy.

**Conclusions:**

Antenatal anaemia, iron deficiency, and CMD have a negative impact on subsequent infant motor development. These findings highlight the need to improve the quality of antenatal care when developing interventions for pregnant women that aim to optimise early childhood development in low- and middle-income countries.

## Background

The major developmental domains in infancy (the period from birth to 12 months of age) are motor, physical, cognitive, and social-emotional [1]. Among the four domains, motor development has received the least attention from researchers. The effects of motor development during infancy on adult functioning are not well-understood, and the crucial role of motor development on the other developmental domains is under-recognised [2].

There is increasing recognition that antenatal maternal health, both physical and mental, is an important determinant of development in infancy with potential persistence of developmental delays or deficits, into adulthood [3]. There are two proposed mechanisms. The first, “foetal programming” has been described as a process by which a stimulus or insult *in utero* caused by a maternal health problem has a long-lasting or permanent effect on foetal physiological functions that render the brain or body vulnerable to developmental delay and/or illnesses later in life [4,5]. The second is that antenatal maternal health problems can increase the risk of adverse pregnancy outcomes including preterm birth and low birthweight [6,7]. Adverse pregnancy outcomes are well-established determinants of developmental delays and disability in children [8]. Therefore, they can mediate the effects of antenatal maternal health problems on children’s developmental outcomes.

Anaemia, characterised by a reduction in haemoglobin (Hb) concentration and the subsequent impairment in the capacity to transport oxygen, has multiple causes including genetic, such as haemoglobinopathies; infections, such as hookworm and malaria; and nutritional including deficiencies of iron, folate and Vitamins C, A and B12 [9]. In pregnant women, anaemia is defined as Hb less than 11 g/dL and severe anaemia Hb less than 7 g/dL [10]. The global prevalence of anaemia in pregnant women is 38% with the highest rates in Central and West Africa (56%) and South Asia (52%) [11]. Iron deficiency is the main cause of anaemia and is thought to account for roughly half of anaemia. However, the proportion of anaemia attributable to this cause varies from place to place depending on the prevalence of other causes (e.g. < 45% in children and non-pregnant women in sub-Saharan African and South Asia to 70% in children and pregnant women in high income countries) [11].

Iron deficiency and anaemia are reported to be related to low birthweight and preterm birth [12,13], low child cognitive development [14,15], and diminutions in neonatal motor maturity [16] in low- and middle-income countries. The effects of iron deficiency and anaemia are rarely separated in existing studies because the common measure of iron deficiency used is a maternal Hb level less than 11 g/dL, which actually reflects the status of anaemia. Low Hb can be used as a proxy indicator of iron deficiency anaemia in a population but cannot be an indicator to detect iron deficiency. However, there is limited evidence in the existing literature for the effects of antenatal iron deficiency and/or anaemia on infant development in general, and infant motor development in particular.

Common mental disorders (CMD), which include depression and anxiety, are prevalent among pregnant women in low- and lower-middle income countries [17]. A number of studies have found an association between maternal antenatal CMD and poor pregnancy outcomes, in particular, premature birth and low birthweight [18,19]. Several studies have demonstrated that antenatal CMD increases the risk of difficult infant temperament and problems with early social engagement [20-26]; and it is suggested to have a negative association with cognitive ability [27,28]. However, DiPietro et al. [29] found that antenatal anxiety and depression were associated with better mental development in children aged 24 months. A study in Ethiopia reported no association between symptoms of CMD in mothers in the third trimester of pregnancy and infant developmental domains including cognitive, language, and motor at 12 months of age [30]. Nasreen et al. [31] did not find any effect of antenatal depression on motor development in infants six to eight months old. Overall, the evidence of association between antenatal CMD and infant development is conflicting. It is possible that the lack of consensus is because none of the existing studies controlled for the important potential confounding factors of antenatal micronutrient deficiencies.

In Vietnam, anaemia, iron deficiency, and common mental disorders are the main public health problems among pregnant women. According to the National Survey on Nutrition 2010, approximately 37% of pregnant women have iron deficiency anaemia (Haemoglobin < 11 g/dL) [32]. The prevalence of anaemia in pregnant women is reported to be up to 53% in some very poor areas [33]. Studies have consistently provided evidence for high prevalence of CMD in women during pregnancy and after childbirth, in particular in the least well-resourced rural areas [34-36].

**Figure 1:**
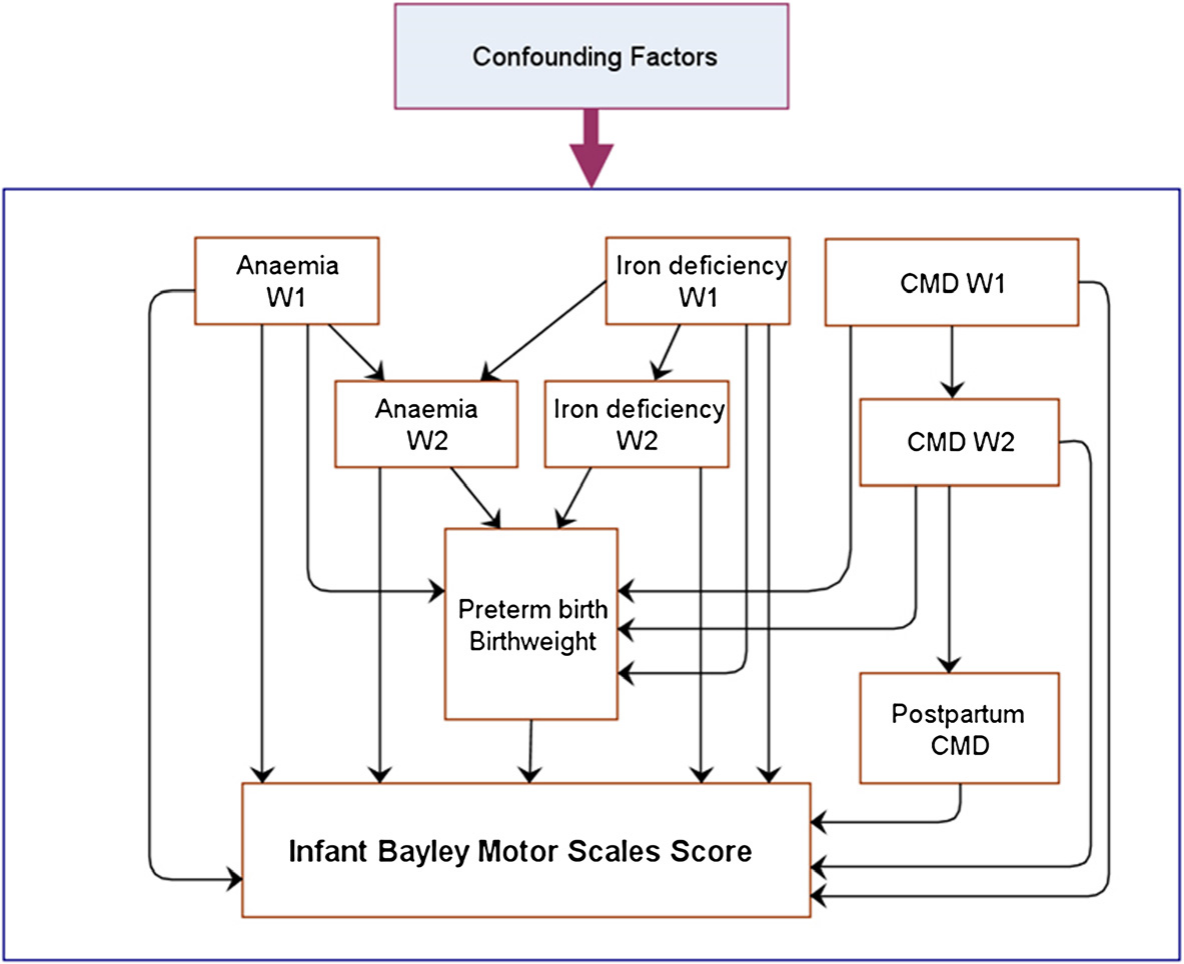
**Hypothesised model of the relationship between maternal antenatal risk factors and infant Bayley Motor Scales score.** Single-headed solid arrows represent the direction of the relationships. CMD: Common mental disorders. W1: Wave 1 (early pregnancy). W2: Wave 2 (late pregnancy).

The aims of this study were to examine the direct and indirect effects of the antenatal risk factors of anaemia, iron deficiency, and CMD on motor development of six month old infants in rural Vietnam. The hypothesised model of the effects, which was derived from existing international and local evidence, is presented in Figure 1. In this model, we postulated that maternal antenatal iron deficiency (W1 and W2), anaemia (W1 and W2), and CMD (W1 and W2) would affect infant motor development at six months via both direct and indirect pathways. The hypothesised direct pathway was that the exposures could cause adverse conditions in utero which affect foetal development and lead, via ‘programming’, to lasting changes in infant development in general and in motor function in particular [4,5,14-16,20-22,28]. The first hypothesised indirect pathway was that the exposures could adversely affect the infant outcomes via lower birthweight and preterm birth [6-8]. The second was that maternal postpartum CMD, which is predicted by antenatal CMD [17,37], can have an adverse effect on infant motor development via less responsive and sensitive caregiving [3]. Birthweight, preterm birth, and postnatal CMD were included in this model as the main mediators. Potential confounders including demographic characteristics and other psychosocial factors that could affect each aspect of the hypothesised model and had to be included [17,30,31,38-42].

## Methods

### Study design, setting, and participants

This investigation is part of a prospective population-based study that followed a systematically-recruited cohort of women from pregnancy to six month postpartum in Ha Nam, a rural province in the Red River Delta, in northern Vietnam.

Ha Nam has a population of 0.8 million inhabitants and is classified as a middle-income province in Vietnam, but by world standards is substantially disadvantaged. The average annual per capita income in 2011 was USD800. Most pregnant women attend at least one antenatal health check and give birth at a health service. Currently iron supplements are not provided free to pregnant women in this area.

Participants were recruited by a two-stage sampling procedure. First, a total of 50 communes were selected randomly from the list of 104 rural communes in Ha Nam using the ‘sample’ command in Stata version 11 (StataCorp LP, College Station, Texas, USA) by an independent statistician. Second, all women pregnant with a single foetus and between 12 and 20 weeks gestation living in the selected communes during the enrolment period (December 2009 to January 2010) were eligible and invited to participate. Women with a known multiple gestation pregnancy or who had a multiple birth were excluded at enrolment or during the study.

### Data sources

#### Infant motor development (collected at W4)

Infant motor development was assessed by direct administration of the Bayley Scales of Infant and Toddler Development 3rd Ed, Fine and Gross Motor Scales (BSID-M) [43] to the infants when they were six months old. The BSID Fine Motor Scale consists of 66 items and Gross Motor Scale 72 items for infants and young children aged from 1 to 42 months. Fine motor items include capacity to gaze at and follow an object, control hand movements, including keeping hands open, reaching for an object with one hand, grasping a block, holding a small piece of food, transferring an object from hand to hand, lifting a cup by the handle, turning the pages of a book, and grasping a crayon or pencil using a palmar grasp (whole hand, fisted). The 35 gross-motor items include being able to control the head, rolling from side to side and from lying on the back to lying on the front, elevating trunk while prone, sitting either with or without support, moving from sitting to hands and knees, crawling, standing with support, and raising self from sitting to a standing position. The administration of each sub-scale is stopped when the infant is unable to do five consecutive items of that sub-scale.

The BSID-M was translated from English into Vietnamese and back translated by a group of bilingual psychologists and health researchers. There are as yet no data available on the validation of the BSID in Vietnam. However, our group has pilot-tested the scale and found it to be comprehensible and meaningful in rural Vietnam. BSID-M scores were highly correlated with BSID cognitive scores (Pearson’s r = 0.64), but not with BISD social-emotional scores (r = 0.12). In the original validation studies conducted in USA, the correlation coefficient of BSID-M scores with BSID cognitive score for infants at 6 months was 0.62 and with BSID social-emotional scores was 0.29, the internal reliability coefficient of BSID for infants at 6 months was 0.90, and the test-retest reliability coefficient was 0.83 [44].

The total raw scores of the BSID-M were converted to composite scores based on the infant’s age in weeks following the guidelines of the BSID Manual [45].

#### Birth outcomes

The first day of the last normal menstrual period (W1) and date of birth (W3) were collected by maternal reports to calculate the gestational age at birth. Infant birthweights were collected by maternal reports at W3 and, when the mother did not remember or was uncertain, were verified against the birth records at the health facility. In every health facility, birthweight was measured immediately after birth (usually within the first hour).

#### Biological data (W1, W2)

Maternal haemoglobin (Hb) was evaluated from a finger prick blood sample using a haemoglobinometer (HemoCue AB, Angelholm Sweden) in the field. Samples of venous blood (3-mL) were taken from women who consented to provide them and centrifuged to harvest serum, frozen in a field freezer and transported in a cold chain to the laboratory of Alfred Pathology Services, Alfred Health, Australia. Serum ferritin was evaluated by Chemiluminescent Microparticle Immuno Assay performed on the Archicentre ci62000 instrument (Abbott, Illinois, USA). Criteria for anaemia was Hb < 11 g/dL [10] and iron deficiency was serum ferritin < 15 ng/mL or < 30 ng/mL in the presence of infection (C-reactive protein > 5 mg/L) as recommended by WHO [46,47].

A spot urine sample was obtained, frozen, and transported to the laboratory of the National Hospital of Endocrinology in Hanoi to determine urine iodine concentrations by means of the Sandell-Kolthoff reaction [48].

#### Maternal mental health status

Symptoms of CMD were assessed at all four waves by the Edinburgh Postnatal Depression Scale-Vietnam Validation (EPDS). The 10-item EPDS yields scores from 0 (no psychological symptoms) to 30 (severe psychological symptoms) [49,50]. The EPDS had been validated against psychiatrist-administered Structural Clinical Interviews for DSM IV diagnoses to establish local cut off scores for women who were pregnant or had recently given birth, including in this province. Internal reliability is 0.75 (95%CI, 0.71–0.78) and scores ≥ 4 detect clinically significant symptoms with a sensitivity of 70% and specificity of 73% [50].

#### Other potential factors of the infant outcome

The following potential factors might influence the infant outcome were assessed:

● Maternal age, marital status, educational level, and occupational status were collected by study-specific questions at W1 [34].

● Household economic status was assessed at W1 by the World Bank method which calculates a Household Wealth Index from information about 17 household characteristics, services and durable assets [51]. The lower the index is the poorer the household is.

● Maternal height was measured with a portable Shorr board (Shorr productions, USA) at W1 and validated at W2.

● Use of iron supplements was assessed at W2 in two questions about whether iron supplements had or had not been taken during the index pregnancy (1: yes; 0: no), and the total duration of use (gestational ages of starting and stopping taking supplements, duration of any temporary cessations of taking supplements).

● Violence: Experiences of intimate partner violence were assessed with the pregnancy section of the WHO Multicountry study on Domestic Violence survey [52], which identifies physical and sexual violence, and emotional abuse. Data were collected at W1, W2 and W4.

● Reproductive health history including parity, history of spontaneous abortions, foetal or neonatal deaths; and whether or not the pregnancy was welcome were collected by study-specific questions at W1

● Coincidental life adversity: was assessed at every all four waves in a single question: *Apart from your pregnancy are there other experiences or aspects of your life that are worrying*?

● Breastfeeding (W4): study-specific structured questions were used to assess whether or not the infant was being breastfed and whether the mother thought that she had sufficient milk for her baby’s needs.

● Infant weight and length (W3 and W4): Infant weight was measured by the Seca 876 Scale (Seca, UK) which first measures maternal weight and second measures the weight of the infant when held in her arms. Infant length was measured with a portable Shorr board (Shorr productions, USA). Weight-for-age Z scores, length-for-age Z scores, and weight-for-length Z scores were calculated by WHO Anthro Version 3.2.2 (WHO 2011). Length-for-age Z scores were used in the analyses because this indicator is not affected by temporary factors such as acute illnesses.

### Procedure

Data collection was conducted at four time points between December 2009 and March 2011. The first (W1) was when the women were recruited and the second (W2) when participants were at least 28 weeks gestation. After childbirth re-assessments of mothers and infants were conducted when the babies were aged about 8 weeks (W3) and about 6 months (W4). Data were collected by face-to-face structured individual interviews conducted in private rooms at commune health centres. The fine motor subscale and gross motor subscale of the BSID were administered to babies in a different room set up to be infant-friendly with a soft clean mat as floor covering and access to soap and water to wash the toys and equipment. All assessments were undertaken by trained, experienced and supervised health research staff and psychologists of the Research and Training Centre for Community Development. Prior to data collection, a pilot study was conducted with 30 mother-infant pairs to check the acceptability and comprehensibility of the data sources used in this study and to standardise data collection procedures.

Approvals to conduct the study were provided by the Ha Nam Provincial Health Department Ethics Committee, the Vietnam Medical Association Ethics and Scientific Committee and the University of Melbourne’s Health Sciences Human Research Ethics Committee. All participants were given an oral and written plain language description of the study and either signed a consent form, or those who could not write provided a thumbprint or verbal consent witnessed by an independent observer.

### Statistical analyses

Path analyses were performed to test the hypothesised model. The main infant outcome was BSID Motor development score at six months of age. The mediators were preterm birth (gestational age < 37 weeks), birthweight, and maternal postpartum CMD (met the criteria of CMD clinically significant symptoms at W3 and/or at W4). Potential confounders were added into the models where appropriate. Two path models were tested. In the first model, antenatal exposures were examined as binary scales, namely anaemia (W1 and W2), iron deficiency (W1 and W2), and CMD (W1 and W2) as in the hypothesised model. In the second model, the antenatal exposures were examined as continuous scales, namely Hb, serum ferritin (log2 transformed to be normally distributed), and EPDS scores at W1 and W2. The first model is easier to interpret, while the second using continuous scales of the exposures maximises the use of data and provides evidence, when available, of dose–response relationships.

The path models were estimated using weighted least-squares and a diagonal weight matrix with standard errors and mean- and variance adjusted chi-square test statistics that use a full weight matrix with pairwise deletion which are recommended for models combining binary and continuous outcomes. Path model coefficients can be interpreted as linear regression coefficients for the paths to continuous outcomes (i.e. BSID-M scores and birthweight). Model coefficients of the paths to binary outcomes (e.g. postpartum CMD) are odds ratios which were derived from original probit regression coefficients for more straightforward interpretation [53]. Criteria to evaluate the good fit of the path model to the observed data are Chi-Square Test of Model Fit with p values greater than 0.05, Root Mean Square Error Of Approximation (RMSEA) with values less than 0.05, and Tucker-Lewis Index (TLI) and Comparative Fit Index (CFI) with values greater than 0.90 [54].

Univariate analyses were performed in Stata 12 (StataCorp LP, College Station, Texas, United States of America, 2011). Path analyses were carried out in Mplus Version 7.1 (Muthén & Muthén, Los Angeles, United States of America, 2013).

## Results

### Sample

In total, 497 of 523 (97%) eligible pregnant women were recruited and provided data at W1. Among them, 79 (15.9%) women were lost to follow-up. Of those, two women had a multiple pregnancy, seven babies were still born, nine women withdrew, 14 were not living in the commune at W2 as they had returned to live with their families-of-origin to give birth and 47 had already given birth when the field team visited to collect W2 data. Finally, 418 women-infant pairs were included in the analyses. Among these 418 women, 40 were missing data of ferritin at W2, 24 were missing Hb at W2, 9 were missing EPDS at W3 and 19 were missing EPDS at W4. A pairwise deletion approach was used to manage these missing data.

There were no differences in the sociodemographic and psychological characteristics of the women included in the analyses and those who were excluded because no data were available for at least one of the follow-up waves (see Table 1). Distributions of Hb and ferritin in early and late pregnancy are presented in Table 2. At about 8 weeks postpartum, most mothers (84.1%) perceived that they had sufficient breast milk to meet the infant’s needs. However, complementary foods were introduced early and none of the infants were breastfed exclusively for the first 26 weeks of life.

**Table 1:** Social-demographic and psychological characteristics of 418 mothers who were included in analyses and 79 women who were not included

**Characteristic**	**Included**	**Not included**	**p-value**
Mother age (years), mean [SD]	26.1 [4.8]	25.8 [5.2]	0.55
Education level, No. (%)			
Partial or complete primary school (Grades 1–5)	77 (18.4)	15 (18.9)	0.39
Secondary school (Grades 6–9)	223 (53.4)	37 (46.9)	
High school (Grades 10–12)	50 (12.0)	8 (10.1)	
Any post-secondary education	68 (16.3)	19 (24.1)	
Occupation, No. (%)			
Farmer	191 (45.7)	31 (39.2)	0.76
Factory, handcraft worker or retailer	131 (31.3)	27 (34.2)	
Government or private officer	50 (12.0)	11 (13.9)	
Not currently engaged in income-generating activity	46 (11.0)	10 (12.7)	
Household wealth index, mean [SD]	−0.01 (1.8)	0.09 (2.1)	0.61
Welcome pregnancy, No. (%)	369 (88.3)	69 (87.3)	0.84
Maternal haemoglobin (g/dL ) – Wave 1, mean [SD]	11.9 (1.2)	11.8 (0.9)	0.91
Maternal ferritin (ng/mL) – Wave 1, median {interquartile range}	64 {39–108}	63 {37–101}	0.55
Maternal height (cm), mean [SD]	153.3 [4.6]	153.2 [4.7]	0.84
Common mental disorders symptoms*, No. (%)			
Wave 1	167 (40.0)	38 (48.1)	0.18
Wave 2	106 (28.0)	N/A	
Wave 3	41 (10.8)^a^	N/A	
Wave 4	47 (12.4)^b^	N/A	

*Common mental disorders symptoms: EPDS-V score ≥ 4; Wave 1: 12–20 weeks of gestation; Wave 2: 32+ weeks of gestation; Wave 3: 8 weeks postpartum; Wave 4: 6 months postpartum;^*a*^*Missing 9 cases;*^*b*^*Missing 19 cases.*

**Table 2:** Maternal antenatal anaemia and iron deficiency of 418 mothers

**Characteristic**	**Early pregnancy (Wave 1)**	**Late pregnancy (Wave 2)**
Haemoglobin (g/dL), mean [SD]	11.9 [1.2]	11.9 [1.5]
Anaemia*, No. (%)	90 (21.5)	96 (24.4)^a^
Ferritin (ng/mL), median {interquartile range}	64 {39–108}	15 {10–27}^b^
Iron deficiency**, No. (%)	17 (4.1)	182 (48.2)

*Anaemia: Haemoglobin < 11 g/dL; ** Iron deficiency: Ferritin < 15 ng/mL;^*a*^*Missing 24 cases;*^*b*^*Missing 40 cases.*

### Birth outcomes and infant motor development

Overall, the mean birthweight was 3.15 kg (SD of 0.40 kg) and 6.3% of the 418 infants had a low birthweight (less than 2.5 kg at birth). The mean gestational age at birth was 39.2 weeks (SD of 2.6) and 14.6% of the infants were born at less than 37 complete weeks of gestation.

The composite infant BSID-M scores were distributed approximately normally at 6 months with a mean score of 95.5 (SD of 15.2, a range from 55 to 142), which was significantly lower than the reference population mean of 100 (SD of 15.0, a range from 55 to 145).

### Path models predicting infant motor development

**Figure 2:**
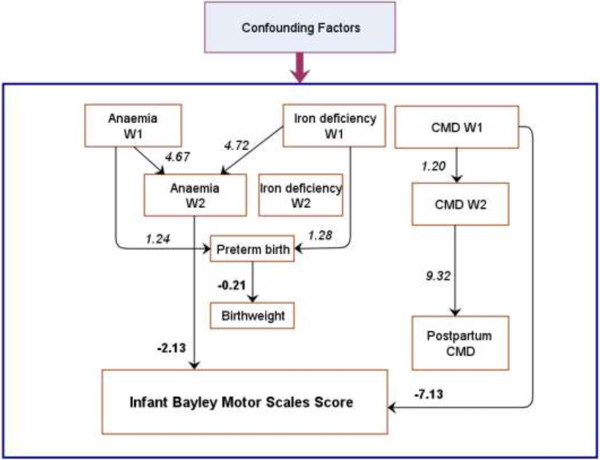
**Path analysis predicting Infant Bayley Motor Scales score by binary antenatal predictors (Model 1).** For more details see Additional file 1. Single-headed solid arrows represent the direction of statistically significant paths. **Bold** path coefficients are the linear regression coefficients. Coefficients in *italics* are converted odds ratios. CMD: Common mental disorders. W1: Wave 1 (early pregnancy). W2: Wave 2 (late pregnancy).

**Figure 3:**
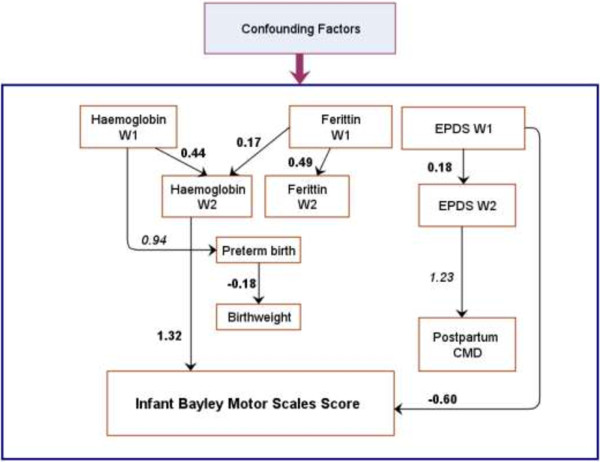
**Path analysis predicting Infant Bayley Motor Scales score by continuous antenatal predictors (Model 2).** For more details see Additional file 2. Single-headed solid arrows represent the direct of the statistically significant paths. **Bold** path coefficients are the linear regression coefficients. Coefficients in *italics* are converted odds ratios. CMD: Common mental disorders. EPDS: Edinburgh Postnatal Depression Scale score. W1: Wave 1 (early pregnancy). W2: Wave 2 (late pregnancy).

The main paths of the two models predicting infant BSID-M score are presented in Figures 2 and 3. Details about the two models are provided in Additional file 1 and Additional file 2. Fitting indices of model 1 (Chi-Square Test of Model Fit with p = 0.36; RMSEA = 0.01; CFI = 0.98; and TLI = 0.97) and model 2 (Chi-Square Test of Model Fit with p = 0.89; RMSEA < 0.01; CFI = 1.0; and TLI = 1.0) indicate that the two models fit the data very well.

In model 1, the hypothesised direct and indirect pathways from maternal antenatal anaemia (W1 and W2), iron deficiency (W1 and W2), and CMD (W1 and W2) to infant BSID-M at 6 months were tested simultaneously. Two of these were statistically significant: CMD at W1 directly decreased the infant outcome by 7.13 points (95% CI, 3.13 to 11.13), about half a standard deviation, and anaemia at W2 directly decreased the infant outcome by 2.61 points (95% CI, 0.57 to 4.65). Other significant paths in Model 1 were that iron deficiency at W1 and anaemia at W1 increased the risk of anaemia at W2; and anaemia at W2 and iron deficiency at W1 were associated with a higher risk of preterm birth. Preterm birth significantly decreased infant birthweight. However, the birth outcomes (preterm birth and birthweight) were not associated with infant BSID-M score. While significant pathways from CMD at W1 and W2 to postpartum CMD were found, postpartum CMD was not associated with the infant outcome.

Model 2 confirms that a higher EPDS score (indicating worse mental health status) at W1 was directly associated with a lower infant BSID-M score at six months of age (regression coefficient of −0.60, 95% CI −1.07 to −0.13). Haemoglobin levels at W2 were associated positively with the infant outcome (regression coefficient of 1.32, 95% CI 0.30 to 2.34). As in Model 1, other hypothesised direct and indirect pathways from maternal haemoglobin (W1), ferritin (W1 and W2), and EPDS score (W2) to infant BSID-M at 6 months were not statistically significant.

Some potential confounders included in the two models were associated significantly with the outcome and hypothesised mediators. Infants of primiparous mothers had lower infant BSID-M scores, while infants of mothers who regarded themselves as having sufficient breastmilk for their baby’s needs had higher scores. Experience of intimate partner violence, suffering coincidental life adversity, having a low education level, and the main occupation as a farmer increased the risk of CMD at both ante- and postnatal periods. Mother’s height and household wealth were positively associated with higher infant birthweight, while nulliparity was associated with lower birthweight. Nulliparity and longer duration of taking iron supplements were associated with a lower risk of anaemia in late pregnancy (see Additional file 1 and Additional file 2).

## Discussion

This study, to our knowledge, is the first ever examination of the simultaneous effects of maternal anaemia, iron deficiency and CMD during pregnancy on infant motor development. These antenatal biological and psychological data were assessed twice (early and late pregnancy) and the main outcome, infant motor development, was assessed by the gold standard Bayley Scales of Infant and Toddler Development 3rd Ed, Fine and Gross Motor Scales. Our data indicate that elevated symptoms of CMD in early pregnancy and lower haemoglobin levels in late pregnancy are significantly related to lower infant BSID-M scores at six months of age. The magnitudes of the effects are clinically significant.

Hernández-Martínez et al. [16] found in Spain that iron deficiency in the third trimester predicted neonatal motor performance and Tamura et al. [15] that low cord serum ferritin concentrations were associated with poor fine-motor skills at 5 years old, In contrast we did not find a direct relationship between maternal antenatal iron deficiency or ferritin concentrations and infant motor development at 6 months of age. It is possible that this is because the prior studies did not control for anaemia, which correlates highly with iron deficiency.

Our data indicate that anaemia in late pregnancy is associated with infant motor development. This finding is consistent with Chang’s [14] investigation of 850 women and their children in China which found that third trimester anaemia (Hb < 11 g/dL) was associated with worse motor development among two year old children. Hernández-Martínez et al. [16] demonstrated that it is not the first or second trimesters but the third trimester that is the critical period of exposure for the adverse effects of iron deficiency on neonatal motor skills. Using path analysis, an advanced statistical method, we could test both indirect and direct pathways simultaneously. We also found that there was no direct effect of anaemia and iron deficiency in early pregnancy on infant BSID-M scores, but we showed that both those conditions affected the outcome indirectly via anaemia in late pregnancy. This finding is novel and suggests that anaemia and iron deficiency in early pregnancy are also essential to infant developmental outcomes.

There are only two prior investigations of the relationship between antenatal CMD and infant motor development [30,31]. Both of those were conducted in low- and middle-income settings and measured antenatal CMD only during the third trimester. Our findings are consistent with their conclusion that there is no significant association between CMD in late pregnancy and infant motor development. Our data indicate however, that it is CMD in early, but not late pregnancy, which is negatively associated with infant motor development. While this requires confirmation in further research, our data suggest that the period from 12 to 20 gestational weeks may be the critical time for the adverse effect of maternal antenatal CMD on infant motor development. Neither of the prior studies collected data in early pregnancy and so they were unable to investigate this relationship.

The mechanisms of the effects of iron deficiency/anaemia and CMD during gestation on infant motor function in particular and infant development in general were not determined explicitly and so we have to speculate what these might be. Maternal antenatal CMD including depression and anxiety can lead to elevations of activity in the HPA axis, which increases levels of cortisol, a major stress hormone, and placental corticotropin-releasing hormone (CRH) [55,56]. Maternal cortisol can pass through the placenta and may account for about 40% of the variation in foetal concentrations [57,58]. Placental CRH is released into both mother and foetus and can also act to release cortisol in the foetus [59]. Exposure to high levels of cortisol may lead to foetal adjustments (foetal programing) that cause long-lasting changes in physical and neurological functions and, potentially, increase vulnerability to developmental delays in each domain [60]. Interestingly, iron deficiency and anaemia can also elevate the release of maternal cortisol and placental CRH to cause an increase of cortisol level in the foetus through increasing norepinephrine concentrations [61,62]. Data of this study could not confirm the postulate that iron deficiency/anaemia and CMD during pregnancy affect infant development through increasing cortisol level in the foetus, but support the relationship between these antenatal exposures and infant motor development at 6 months of age.

Regardless of the causal mechanism these data indicate that there is an adverse impact of these exposures on both fine and gross motor development. The deficits in gross motor skills are reflected in being slower to meet major milestones like sitting, crawling and standing. Fine motor skills are essential to exploring the environment through being able to hold and explore objects and to experiencing a sense of agency through actions that lead to outcomes, e.g. shaking a rattle and hearing the sound or turning the pages of a book to see a new image. These in turn are fundamental to the stimulation that underpins cognitive and language development.

## Conclusions

This study has several limitations. First, we acknowledge that several potential important antenatal factors which might influence infant development were not, because of feasibility constraints in this low income setting, considered in this study, including deficiencies in zinc or vitamins B or D and environmental toxins. Antenatal smoking and alcohol use are also relevant exposures, but we have established that these are exceptionally rare among women in this study setting. Second, maternal CMD was detected by EPDS, a screening tool, which does not yield diagnoses and does not distinguish between depression and anxiety. However, in this setting the EPDS clinical cut-off score that we used has a high level of sensitivity (70%) and specificity (73%) when validated against a diagnostic psychiatric interview to detect CMD including depression and anxiety in perinatal women [50]. Third, ferritin concentration, that is the only nutritional biomarker used in this study to assess iron status, is of limited usefulness in diagnosing iron deficiency during pregnancy as concentrations fall during late pregnancy and rise in response to inflammation [63]. We have used several mitigation strategies including (1) two models were used to test ferritin concentration as both a continuous and a binary (using a cut-off to determine iron deficiency) variable and the results were consistent, and (2) the cut-off of iron deficiency was adjusted for the presence of inflammation as suggested by WHO [47]. Finally, that BSID had not been validated in Vietnam limited the potential for comparisons between infant motor development outcomes in this study and other populations. However, each item of the scale was reviewed and pilot tested carefully and this permits us to make comparisons between groups within the sample with confidence. This study is to our knowledge the first to combine assessment of antenatal anaemia, iron deficiency, and psychosocial risks in a single investigation. The data confirm that anaemia, iron deficiency, and CMD during pregnancy are prevalent in rural Vietnam. These antenatal risks are related to lower infant motor development. CMD in early pregnancy and anaemia in late pregnancy are directly related to lower infant motor development at six months of age, while anaemia and iron deficiency at early pregnancy were indirectly associated with the infant outcome via increasing risk of anaemia at late pregnancy. Our study suggests that interventions to promote infant development should address these antenatal factors explicitly. Future studies may focus on investigating the effect on infant motor development of interventions that address these antenatal risks simultaneously.

## Abbreviations

BSID-M: Bayley Scales of Infant and Toddler Development 3rd Ed, Fine and Gross Motor Scales; CFI: Comparative fit index; CI: Confidence interval; CMD: Common mental disorders; CRH: Corticotropin-releasing hormone; EPDS: The Edinburgh Postnatal Depression Scale-Vietnam Validation; Hb: Maternal haemoglobin; RMSEA: Root mean square error of approximation; SD: Standard deviation; TLI: Tucker-Lewis Index; W1: Wave One: baseline survey conducted when the women were 12–20 gestational weeks; W2: Wave Two: second survey when participants were at least 28 gestational weeks; W2: Wave three: third survey conducted when the babies were 8 weeks; W4: Wave four: fourth survey conducted when the babies were 6 months; WHO: World Health Organization.

## Competing interests

The authors declare that they have no conflicts of interests.

## Authors’ contributions

TDT secured the competitive grant, participated in the design of this study, conducted training of the data collectors, coordinated data collection and data management, performed data analysis, and drafted the manuscript. TT participated in the design of this study and secured the grant. JAS participated in data analysis. HTT, TTN, and SH participated in data collection. TD and BB secured the grant and contributed to the design of this study. JF secured the grant, participated in the design of this study, data collection, and writing the first draft of the manuscript. All authors contributed to critically revising, read, and approved the final manuscript.

## Pre-publication history

The pre-publication history for this paper can be accessed here:

http://www.biomedcentral.com/1471-2393/14/8/prepub

## Supplementary Material

Additional file 1 Path analysis predicting Bayley Scales of Infant and Toddler Development – Motor Scales (BSID-M) score by binary antenatal predictors (Model 1). Click here for file

Additional file 2 Path analysis predicting Bayley Scales of Infant and Toddler Development – Motor Scales (BSID-M) score by continuous antenatal predictors (Model 2). Click here for file
