# Supplementary material for: Infant motor development in rural Vietnam and intrauterine exposures to anaemia, iron deficiency and common mental disorders: a prospective community-based study
Source: BMC Pregnancy Childbirth. 2014 Jan 8;14:8. doi: 10.1186/1471-2393-14-8 (PMC3890590; doi:10.1186/1471-2393-14-8)
Supplement: Additional file 1 — Path analysis predicting Bayley Scales of Infant and Toddler Development – Motor Scales (BSID-M) score by binary antenatal predictors (Model 1). [file 1471-2393-14-8-S1.docx]

Additional 1 – Path analysis predicting Bayley Scales of Infant and Toddler Development – Motor Scales (BSID-M) score by binary antenatal predictors (Model 1)

|  | | Path coefficient | | 95%CI | |  |  |
| --- | --- | --- | --- | --- | --- | --- | --- |
| **Iron deficiency W2** | | Odds ratio | |  |  |  |  |
| Iron deficiency W1 | | 2.17 | | 0.87 | 5.42 |  |  |
| 75% highest household wealth index | | 1.10 | | 0.86 | 1.40 |  |  |
| Length of time taking iron supplements (%) | | 0.99 | | 0.99 | 1.00 |  |  |
| Complete year 9 or higher | | 1.00 | | 0.82 | 1.23 |  |  |
| Maternal age (year) | | 0.91 | | 0.73 | 1.13 |  |  |
| Primiparity | | 0.70 | | 0.44 | 1.11 |  |  |
| Maternal occupation (Farmer) | | 1.02 | | 0.97 | 1.07 |  |  |
|  | |  | |  |  |  |  |
| **Anaemia W2** | | Odds ratio | |  |  |  |  |
| Iron deficiency W1 | | 4.72 | | 2.12 | 10.54 |  |  |
| Iron deficiency W2 | | 1.40 | | 0.86 | 2.30 |  |  |
| Anaemia W1 | | 4.67 | | 2.25 | 9.68 |  |  |
| Primiparity | | 0.43 | | 0.20 | 0.91 |  |  |
| Length of time taking iron supplements (%) | | 0.98 | | 0.97 | 0.99 |  |  |
| Complete year 9 or higher | | 2.20 | | 0.83 | 4.38 |  |  |
| Maternal age (year) | | 0.74 | | 0.41 | 1.35 |  |  |
| 75% highest household wealth index | | 0.76 | | 0.37 | 1.56 |  |  |
| Maternal occupation (Farmer) | | 0.91 | | 0.78 | 1.07 |  |  |
|  | |  | |  |  |  |  |
| **CMD W2** | | Odds ratio | |  |  |  |  |
| CMD W1 | | 1.20 | | 1.02 | 1.41 |  |  |
| Primiparity | | 1.02 | | 0.86 | 1.21 |  |  |
| Experience of childhood abuse | | 1.21 | | 0.96 | 1.51 |  |  |
| Experience of lifetime intimate partner violence | | 1.21 | | 1.00 | 1.46 |  |  |
| Complete year 9 or higher | | 1.09 | | 0.91 | 1.29 |  |  |
| Maternal age (year) | | 0.89 | | 0.76 | 1.03 |  |  |
| 75% highest household wealth index | | 0.95 | | 0.79 | 1.14 |  |  |
| Coincidental life adversity | | 1.57 | | 1.27 | 1.94 |  |  |
| Welcome pregnancy | | 0.89 | | 0.71 | 1.10 |  |  |
| History of miscarriage/stillbirth | | 1.20 | | 0.98 | 1.46 |  |  |
| Maternal occupation (Farmer) | | 1.00 | | 0.97 | 1.03 |  |  |
| Affectionate relationship with own mother | | 1.04 | | 0.87 | 1.23 |  |  |
| Affectionate relationship with mother-in-law | | 0.99 | | 0.86 | 1.15 |  |  |
|  | |  | |  |  |  |  |
| **Infant Birthweight (kg)** | | Regression coefficient | |  |  |  |  |
| CMD W2 | | 0.04 | | -0.05 | 0.13 |  |  |
| CMD W1 | | 0.00 | | -0.09 | 0.10 |  |  |
| Iron deficiency W1 | | 0.23 | | -0.09 | 0.64 |  |  |
| Iron deficiency W2 | | 0.05 | | -0.02 | 0.12 |  |  |
| Anaemia W1 | | 0.10 | | -0.01 | 0.20 |  |  |
| Anaemia W2 | | -0.02 | | -0.07 | 0.03 |  |  |
| Maternal height (10 cm) | | 0.15 | | 0.07 | 0.23 |  |  |
| Preterm birth | | -0.21 | | -0.29 | -0.12 |  |  |
| Complete year 9 or higher | | 0.01 | | -0.09 | 0.11 |  |  |
| Maternal age (year) | | 0.02 | | -0.07 | 0.10 |  |  |
| 75% highest household wealth index | | 0.12 | | 0.02 | 0.22 |  |  |
| Primiparity | | -0.17 | | -0.27 | -0.06 |  |  |
| Child sex (Boy) | | 0.05 | | -0.03 | 0.13 |  |  |
| Low UIC | | 0.03 | | -0.04 | 0.10 |  |  |
| Welcome pregnancy | | 0.06 | | -0.06 | 0.17 |  |  |
| Maternal occupation (Farmer) | | 0.00 | | -0.02 | 0.02 |  |  |
|  | |  | |  |  |  |  |
| **Preterm birth** | | Odds ratio | |  |  |  |  |
| CMD W2 | | 1.09 | | 0.94 | 1.27 |  |  |
| CMD W1 | | 1.03 | | 0.88 | 1.21 |  |  |
| Iron deficiency W1 | | 1.28 | | 1.05 | 1.56 |  |  |
| Iron deficiency W2 | | 1.03 | | 0.93 | 1.14 |  |  |
| Anaemia W1 | | 1.24 | | 1.05 | 1.48 |  |  |
| Anaemia W2 | | 0.97 | | 0.90 | 1.05 |  |  |
| Maternal height (10 cm) | | 1.02 | | 0.88 | 1.18 |  |  |
| Complete year 9 or higher | | 0.91 | | 0.75 | 1.10 |  |  |
| Maternal age (year) | | 0.95 | | 0.82 | 1.11 |  |  |
| 75% highest household wealth index | | 0.95 | | 0.81 | 1.11 |  |  |
| Primiparity | | 0.93 | | 0.77 | 1.13 |  |  |
| Child sex (Boy) | | 0.97 | | 0.85 | 1.11 |  |  |
| Low UIC | | 1.05 | | 0.94 | 1.17 |  |  |
| Welcome pregnancy | | 1.08 | | 0.86 | 1.36 |  |  |
| Maternal occupation (Farmer) | | 1.00 | | 0.97 | 1.04 |  |  |
|  | |  | |  |  |  |  |
| **Postpartum CMD** | | Odds ratio | |  |  |  |  |
| CMD W2 | | 9.32 | | 3.82 | 22.76 |  |  |
| CMD W1 | | 1.58 | | 0.81 | 3.07 |  |  |
| Coincidental life adversity | | 5.25 | | 1.96 | 14.02 |  |  |
| Primiparity | | 3.28 | | 1.57 | 6.85 |  |  |
| Experience of childhood abuse | | 2.03 | | 0.82 | 5.05 |  |  |
| Experience of intimate partner violence since childbirth | | 7.46 | | 1.65 | 33.63 |  |  |
| Preterm birth | | 0.58 | | 0.28 | 1.19 |  |  |
| Infant Birthweight (kg) | | 1.61 | | 0.79 | 3.29 |  |  |
| Complete year 9 or higher | | 0.26 | | 0.11 | 0.64 |  |  |
| Maternal age (year) | | 1.42 | | 0.74 | 2.74 |  |  |
| 75% highest household wealth index | | 1.22 | | 0.53 | 2.77 |  |  |
| Child sex (Boy) | | 0.87 | | 0.44 | 1.71 |  |  |
| Welcome pregnancy | | 0.45 | | 0.18 | 1.15 |  |  |
| Maternal occupation (Farmer) | | 1.19 | | 1.01 | 1.41 |  |  |
| Affectionate relationship with own mother | | 0.80 | | 0.34 | 1.92 |  |  |
| Affectionate relationship with mother-in-law | | 1.18 | | 0.58 | 2.41 |  |  |
|  | |  | |  |  |  |  |
| **BSID-M score** | | Regression coefficient | |  |  |  |  |
| CMD W2 | | 3.45 | | -1.84 | 8.74 |  |  |
| CMD W1 | | -7.13 | | -11.13 | -3.13 |  |  |
| Postpartum CMD | | 0.45 | | -1.65 | 2.55 |  |  |
| Infant Birthweight (kg) | | 1.93 | | -1.70 | 5.56 |  |  |
| Iron deficiency W1 | | -2.13 | | -7.56 | 3.30 |  |  |
| Iron deficiency W2 | | -1.24 | | -4.14 | 1.66 |  |  |
| Anaemia W1 | | 2.44 | | -1.26 | 6.14 |  |  |
| Anaemia W2 | | -2.61 | | -4.65 | -0.57 |  |  |
| Primiparity | | -4.24 | | -8.45 | -0.03 |  |  |
| Sufficient breastmilk for infant’s demand | | 7.71 | | 3.22 | 12.20 |  |  |
| Preterm birth | | 2.74 | | -1.16 | 6.64 |  |  |
| Complete year 9 or higher | | 2.59 | | -1.58 | 6.76 |  |  |
| Maternal age (year) | | 0.20 | | -3.21 | 3.61 |  |  |
| 75% highest household wealth index | | -1.07 | | -5.07 | 2.93 |  |  |
| Child sex (Boy) | | -2.17 | | -5.23 | 0.89 |  |  |
| Low UIC | | -1.13 | | -3.82 | 1.56 |  |  |
| Welcome pregnancy | | -1.58 | | -7.79 | 4.63 |  |  |
| Maternal occupation (Farmer) | | -0.38 | | -1.24 | 0.48 |  |  |
| Infant length for age Z-score W3 | | 1.27 | | -0.85 | 3.39 |  |  |
| Infant length for age Z-score W4 | | -1.11 | | -3.46 | 1.24 |  |  |
| **Fit indices** | |  | | **Estimates** | | | |
| χ*^2^*/*df* (p-value) | |  | | 116/137 (0.89) | | | |
| RMSEA (Probability RMSEA <= .05) | |  | | <0.01 (0.99) | | | |
| CFI | |  | | 1.00 | | | |
| TLI | |  | | 1.00 | | | |
