# Supplementary material for: Infant motor development in rural Vietnam and intrauterine exposures to anaemia, iron deficiency and common mental disorders: a prospective community-based study
Source: BMC Pregnancy Childbirth. 2014 Jan 8;14:8. doi: 10.1186/1471-2393-14-8 (PMC3890590; doi:10.1186/1471-2393-14-8)
Supplement: Additional file 2 — Path analysis predicting Bayley Scales of Infant and Toddler Development – Motor Scales (BSID-M) score by continuous antenatal predictors (Model 2). [file 1471-2393-14-8-S2.docx]

Additional file 2 – Path analysis predicting Bayley Scales of Infant and Toddler Development – Motor Scales (BSID-M) score by continuous antenatal predictors (Model 2)

|  | | Path coefficient | | 95%CI | |  |  |
| --- | --- | --- | --- | --- | --- | --- | --- |
| **Ferritin W2** | | Regression coefficient | |  |  |  |  |
| Ferritin W1 | | 0.49 | | 0.42 | 0.56 |  |  |
| 75% highest household wealth index | | -0.16 | | -0.36 | 0.05 |  |  |
| Length of time taking iron supplements (%) | | 0.01 | | 0.00 | 0.01 |  |  |
| Complete year 9 or higher | | -0.01 | | -0.19 | 0.18 |  |  |
| Maternal age (year) | | 0.07 | | -0.09 | 0.23 |  |  |
| Primiparity | | 0.18 | | -0.02 | 0.37 |  |  |
| Maternal occupation (Farmer) | | 0.04 | | -0.13 | 0.21 |  |  |
|  | |  | |  |  |  |  |
| **Haemoglobin W2** | | Regression coefficient | |  |  |  |  |
| Ferritin W1 | | 0.17 | | 0.02 | 0.31 |  |  |
| Ferritin W2 | | 0.01 | | -0.15 | 0.17 |  |  |
| Haemoglobin W1 | | 0.44 | | 0.35 | 0.53 |  |  |
| Primiparity | | 0.36 | | -0.03 | 0.74 |  |  |
| Length of time taking iron supplements (%) | | 0.01 | | 0.01 | 0.02 |  |  |
| Complete year 9 or higher | | -0.35 | | -0.76 | 0.05 |  |  |
| Maternal age (year) | | 0.22 | | -0.11 | 0.54 |  |  |
| 75% highest household wealth index | | -0.04 | | -0.40 | 0.33 |  |  |
| Maternal occupation (Farmer) | | 0.23 | | -0.13 | 0.60 |  |  |
|  | |  | |  |  |  |  |
| **EPDS score W2** | | Regression coefficient | |  |  |  |  |
| EPDS score W1 | | 0.18 | | 0.10 | 0.26 |  |  |
| Primiparity | | 0.24 | | -0.71 | 1.19 |  |  |
| Experience of childhood abuse | | 1.15 | | 0.03 | 2.27 |  |  |
| Experience of lifetime intimate partner violence | | 1.13 | | 0.22 | 2.04 |  |  |
| Complete year 9 or higher | | 0.25 | | -0.67 | 1.17 |  |  |
| Maternal age (year) | | 0.03 | | -0.81 | 0.86 |  |  |
| 75% highest household wealth index | | 0.48 | | -0.45 | 1.42 |  |  |
| Coincidental life adversity | | -0.83 | | -1.59 | -0.06 |  |  |
| Welcome pregnancy | | 0.02 | | -1.02 | 1.06 |  |  |
| History of miscarriage/stillbirth | | 2.19 | | 1.34 | 3.04 |  |  |
| Maternal occupation (Farmer) | | 0.76 | | -0.29 | 1.81 |  |  |
| Affectionate relationship with own mother | | -1.35 | | -2.41 | -0.28 |  |  |
| Affectionate relationship with mother-in-law | | -0.35 | | -1.19 | 0.50 |  |  |
|  | |  | |  |  |  |  |
| **Infant Birthweight** | | Regression coefficient | |  |  |  |  |
| EPDS score W2 | | 0.00 | | -0.01 | 0.01 |  |  |
| EPDS score W1 | | 0.00 | | -0.01 | 0.01 |  |  |
| Ferritin W1 | | -0.04 | | -0.10 | 0.01 |  |  |
| Ferritin W2 | | -0.04 | | -0.10 | 0.02 |  |  |
| Haemoglobin W1 | | -0.01 | | -0.04 | 0.02 |  |  |
| Haemoglobin W2 | | -0.01 | | -0.03 | 0.02 |  |  |
| Maternal height (10 cm) | | 0.15 | | 0.07 | 0.23 |  |  |
| Preterm birth | | -0.18 | | -0.26 | -0.10 |  |  |
| Complete year 9 or higher | | 0.02 | | -0.08 | 0.11 |  |  |
| Maternal age (year) | | 0.01 | | -0.07 | 0.10 |  |  |
| 75% highest household wealth index | | 0.13 | | 0.03 | 0.23 |  |  |
| Primiparity | | -0.15 | | -0.25 | -0.05 |  |  |
| Child sex (Boy) | | 0.05 | | -0.03 | 0.14 |  |  |
| Low UIC | | -0.01 | | -0.04 | 0.03 |  |  |
| Welcome pregnancy | | 0.06 | | -0.06 | 0.18 |  |  |
| Maternal occupation (Farmer) | | -0.04 | | -0.12 | 0.05 |  |  |
|  | |  | |  |  |  |  |
| **Preterm birth** | | Odds ratio | |  |  |  |  |
| EPDS score W2 | | 1.00 | | 0.98 | 1.02 |  |  |
| EPDS score W1 | | 1.01 | | 0.99 | 1.03 |  |  |
| Ferritin W1 | | 0.98 | | 0.91 | 1.05 |  |  |
| Ferritin W2 | | 1.02 | | 0.93 | 1.11 |  |  |
| Haemoglobin W1 | | 0.94 | | 0.89 | 0.99 |  |  |
| Haemoglobin W2 | | 0.99 | | 0.95 | 1.04 |  |  |
| Maternal height (10 cm) | | 1.02 | | 0.88 | 1.19 |  |  |
| Complete year 9 or higher | | 0.90 | | 0.74 | 1.09 |  |  |
| Maternal age (year) | | 0.95 | | 0.82 | 1.10 |  |  |
| 75% highest household wealth index | | 0.95 | | 0.81 | 1.11 |  |  |
| Primiparity | | 0.94 | | 0.78 | 1.13 |  |  |
| Child sex (Boy) | | 0.97 | | 0.85 | 1.12 |  |  |
| Low UIC | | 0.99 | | 0.93 | 1.05 |  |  |
| Welcome pregnancy | | 1.07 | | 0.85 | 1.34 |  |  |
| Maternal occupation (Farmer) | | 1.03 | | 0.88 | 1.20 |  |  |
|  | |  | |  |  |  |  |
| **Postpartum EPDS score** | | Odds ratio | |  |  |  |  |
| EPDS score W2 | | 1.23 | | 1.16 | 1.31 |  |  |
| EPDS score W1 | | 1.07 | | 1.00 | 1.14 |  |  |
| Coincidental life adversity | | 4.91 | | 1.79 | 13.43 |  |  |
| Primiparity | | 3.12 | | 1.54 | 6.35 |  |  |
| Experience of childhood abuse | | 1.99 | | 0.80 | 4.96 |  |  |
| Experience of intimate partner violence since childbirth | | 6.74 | | 1.48 | 30.68 |  |  |
| Preterm birth | | 0.69 | | 0.33 | 1.42 |  |  |
| Infant Birthweight | | 1.87 | | 0.94 | 3.70 |  |  |
| Complete year 9 or higher | | 0.27 | | 0.12 | 0.62 |  |  |
| Maternal age (year) | | 1.44 | | 0.75 | 2.77 |  |  |
| 75% highest household wealth index | | 1.13 | | 0.50 | 2.54 |  |  |
| Child sex (Boy) | | 0.92 | | 0.46 | 1.82 |  |  |
| Welcome pregnancy | | 0.52 | | 0.21 | 1.30 |  |  |
| Maternal occupation (Farmer) | | 2.30 | | 1.13 | 4.70 |  |  |
| Affectionate relationship with own mother | | 0.77 | | 0.33 | 1.79 |  |  |
| Affectionate relationship with mother-in-law | | 1.18 | | 0.59 | 2.37 |  |  |
|  | |  | |  |  |  |  |
| **BSID-M score** | | Regression coefficient | |  |  |  |  |
| EPDS score W2 | | 0.43 | | -0.06 | 0.92 |  |  |
| EPDS score W1 | | -0.60 | | -1.07 | -0.13 |  |  |
| Postpartum EPDS score | | 0.28 | | -1.72 | 2.28 |  |  |
| Infant Birthweight | | 2.17 | | -1.42 | 5.76 |  |  |
| Ferritin W1 | | 0.31 | | -1.96 | 2.58 |  |  |
| Ferritin W2 | | -0.06 | | -2.59 | 2.47 |  |  |
| Haemoglobin W1 | | -0.07 | | -1.21 | 1.07 |  |  |
| Haemoglobin W2 | | 1.32 | | 0.30 | 2.34 |  |  |
| Primiparity | | -2.43 | | -6.51 | 1.65 |  |  |
| Sufficient breastmilk for infant’s demand | | 7.35 | | 2.98 | 11.72 |  |  |
| Preterm birth | | 3.29 | | -0.41 | 6.99 |  |  |
| Complete year 9 or higher | | 1.92 | | -2.18 | 6.02 |  |  |
| Maternal age (year) | | 0.43 | | -3.00 | 3.86 |  |  |
| 75% highest household wealth index | | -0.66 | | -4.60 | 3.28 |  |  |
| Child sex (Boy) | | -2.27 | | -5.39 | 0.85 |  |  |
| Low UIC | | 0.32 | | -1.27 | 1.91 |  |  |
| Welcome pregnancy | | -1.12 | | -7.43 | 5.19 |  |  |
| Maternal occupation (Farmer) | | -2.56 | | -6.26 | 1.14 |  |  |
| Infant length for age Z-score W3 | | 1.27 | | -0.85 | 3.39 |  |  |
| Infant length for age Z-score W4 | | -1.11 | | -3.46 | 1.24 |  |  |
| **Fit indices** | |  | | **Estimates** | | | |
| χ*^2^*/*df* (p-value) | |  | | 142/137 (0.36) | | | |
| RMSEA (Probability RMSEA <= .05) | |  | | 0.01 (0.99) | | | |
| CFI | |  | | 0.98 | | | |
| TLI | |  | | 0.97 | | | |
